# Genomic survey uncovers the emergence of a multidrug-resistant dominant lineage in *Proteus mirabilis* populations

**DOI:** 10.1038/s44259-026-00189-5

**Published:** 2026-03-03

**Authors:** Tiejun Zhang, Hongcheng Wei, Zijing Ju, Qin Wang, Jinpeng Li, Renqiao Wen, Jie Wu, Hongning Wang, Changwei Lei

**Affiliations:** 1 College of Life Sciences, Sichuan University, Chengdu, China; 2 Key Laboratory of Bio-Resource and Eco-Environment of Ministry of Education, Chengdu, China; 3 Animal Disease Prevention and Green Development Key Laboratory of Sichuan Province, Chengdu, China

**Keywords:** Computational biology and bioinformatics, Genetics, Microbiology

## Abstract

*Proteus mirabilis*, a Gram-negative bacterium renowned for its distinctive swarming motility, is a major causative agent of catheter-associated urinary tract infections (CAUTIs) and nosocomial complications. While advances in sequencing technologies have generated extensive genomic data, critical gaps persist in understanding the global population structure and evolutionary drivers of antimicrobial resistance in this pathogen. To address this knowledge gap, we performed a phylogenomic analysis of 1,142 *P. mirabilis* genomes spanning 34 countries and 16 ecological niches. Our investigation identified a dominant multidrug-resistant lineage (Cluster-1) carrying significantly elevated antimicrobial resistance gene burdens, including high-prevalence carbapenemase genes *bla*_KPC-2_ and *bla*_IMP-27_. Bayesian evolutionary dating traced the most recent common ancestor of Cluster-1 to approximately 1910, with subsequent expansion linked to acquisition of the autotransporter gene *agn43* within the PmGRI1 genomic island. Notably, Cluster-1 diversified into two clinically significant subclades: a China-associated branch carrying *bla*_KPC-2_ and a USA-associated branch harboring *bla*_IMP-27_. Functional characterization revealed that *agn43* deletion caused significant attenuation in biofilm formation, heat stress tolerance, and swarming motility. Our findings delineate the emergence of a globally disseminated *P. mirabilis* clone, highlighting the synergistic role of antimicrobial resistance and *agn43*-mediated adaptive traits in its epidemiological success.

## Introduction

*Proteus mirabilis*, a Gram-negative opportunistic pathogen, is a leading cause of catheter-associated urinary tract infections (CAUTIs), particularly in healthcare settings, and is associated with severe complications such as pyelonephritis, urolithiasis, and bacteremia^1,2^. While naturally colonizing the gastrointestinal tracts of humans and animals^2^, its ability to form biofilms and exhibit swarming motility enables persistent colonization of urinary catheters and rapid tissue invasion^3,4^. Alarmingly, *P. mirabilis* exhibits intrinsic resistance to last-resort antibiotics (e.g., colistin and tigecycline), and the global emergence of extended-spectrum β-lactamase (ESBL)-, AmpC-, and carbapenemase-producing strains has further narrowed therapeutic options^5^. Recent studies highlight its role as a potential reservoir for horizontally transmitted resistance genes, exemplified by the *P. mirabilis* Genomic Resistance Island 1 (PmGRI1), which harbors multidrug resistance determinants including carbapenemase gene *bla*_KPC-2_, ESBL gene *bla*_CTX-M-3_ and fluoroquinolone resistance gene *aac(6’)-Ib-cr*^6–10^. In addition, we also discovered the autotransporter gene *agn43* on PmGRI1. *agn43* encodes the Ag43 autotransporter. Ag43 is a modular protein composed of three major domain, namely (i) an N-terminal signal peptide (SP), (ii) a passenger domain, and (iii) a C-terminal transporter^11^. The crystal structure of the functional passenger domain of Ag43 has a unique L-shaped structure that drives the formation of cellular aggregates through a handshake mechanism similar to molecular Velcro^11^. The role of Ag43 in self-aggregation, adhesion, and biofilm formation has been demonstrated in enterohemorrhagic *Escherichia coli* (EHEC) and urinary tract infection *Escherichia coli* UPEC^12,13^. However, the specific functional contribution of *agn43* in *P. mirabilis* remains uncharted, warranting further exploration.

Despite the exponential growth of bacterial genomic data, current knowledge of *P. mirabilis* population structure and evolution remains fragmented, with most studies limited to small-scale genomic analyses or regional isolates. In this study, we integrate a global genomic dataset of 1142 *P. mirabilis* isolates spanning 34 countries and diverse hosts to delineate the population structure, evolutionary history, and genomic determinants of epidemic lineages. Combining phylogenomic dating, comparative genomics, and functional assays, we identify a predominant multidrug-resistant lineage (Cluster-1) that emerged in the early 20th century and subsequently underwent rapid global dissemination. We demonstrate that the PmGRI1-encoded *agn43* gene is one of the drivers of this lineage’s success, enhancing biofilm formation, thermotolerance, and swarming motility, traits critical for hospital persistence and host adaptation.

## Results

### *P. mirabilis* population structure

**Fig. 1:**
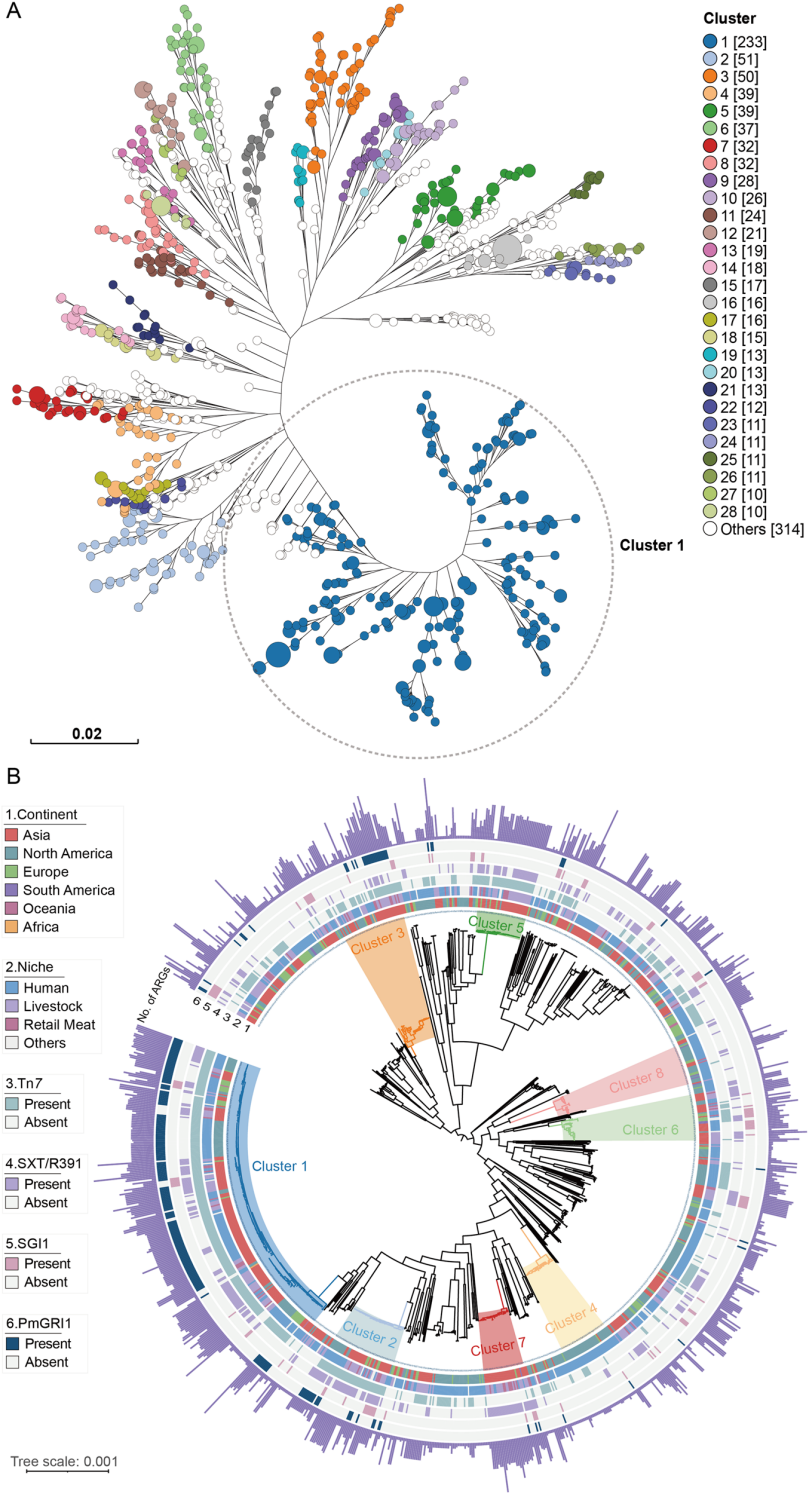
The population structure of 1142 *Proteus mirabilis.* **A** Whole-genome clustering for 1142 *Proteus mirabilis* genomes by PopPUNK. **B** Maximum likelihood tree of 1142 *Proteus mirabilis* core genomes, rooted at the midpoint, the outermost circle shows the number of drug resistance genes, and the inner six circles show the continental origin, ecological niche, and the presence of four mobile genetic elements (Tn*7*, SXT/R391, SGI1, PmGRI1) of the strains. The eight largest clusters are marked in different colors on the evolutionary tree.

To elucidate the population structure, we performed genome clustering using PopPUNK, which resolved 178 clusters (Fig. 1A). Cluster-1 emerged as the most prominent, containing 233 genomes (20% of the total), outnumbering the second cluster (Cluster 2, *n* = 51) by 4.6-fold. The phylogenetic analysis corroborated the clustering results, with Cluster-1 constituting the major lineage (Fig. 1B). Strains within Cluster-1 predominantly originated from humans (63.9%, 59.4% in 1142 isolates), followed by livestock (24.8%, 28% in 1142 isolates), retail meat (7.7%, 7.7% in 1142 isolates), and other sources (3.4%, 4.7% in 1142 isolates). Notably, the PmGRI1 genomic resistance island was significantly enriched in Cluster-1 (71.1%, *p* = 2.7e–83; odds ratio = 28.30, 95% CI: 19.25, 42.10).

To reveal the driving factors for Cluster-1 to become the dominant lineage, we conducted a genome-wide association study (GWAS). A total of 178 genes (including 77 hypothetical protein–encoding genes) were significantly enriched in Cluster-1, encompassing CRISPR-associated genes, multiple insertion sequence families (IS*3*, IS*5*, and IS*66*), the Tn*7* transposon, the autotransporter gene *agn43*, and the toxin–antitoxin module *parDE*, among others. (Supplementary Data 2). It is also worth noting that compared with other lineages, Cluster-1 has a different ubiquinone biosynthesis accessory factor *ubiK*, and the nucleotide similarity with *ubiK* in other lineages is 90.9%.

Given the high prevalence of Tn*7* in *P. mirabilis* (*n* = 593), we extracted 349 complete Tn*7* elements and analyzed their associated ARGs. Individual Tn*7* elements typically carried 1–4 ARGs, although two isolates harbored Tn*7* copies with as many as 18 ARGs. The most common ARG combination was *aadA1–sat2–dfrA1* (*n* = 254). Notably, *aadA1–sat2–bla*_IMP-27_ represented the second most frequent ARG combination (*n* = 51). Isolates carrying this combination were first recovered in the United States in 2016 and were subsequently detected between 2016 and 2022 in both the United States and Canada. Tn*7* elements harboring *bla*_IMP-27_ were widely distributed across 17 clusters, including Cluster-1 (Supplementary Data 4).

SNP distance analysis revealed a median of 4319 SNPs between all isolates, with a range of 0-7529 SNPs (Fig. S1). Within Cluster-1, the median SNP distance was markedly lower (181 SNPs, range: 0-1707 SNPs), indicating a closer genetic relationship among its members. Multilocus sequence typing (MLST) identified 288 STs, of which 138 were novel, with ST135 being the most prevalent and associated with Cluster-1 (Figs. S2, S3, S5).

### The major lineage Cluster-1 genomes carry more antimicrobial resistance genes and rich in virulence gene *agn43*

**Fig. 2:**
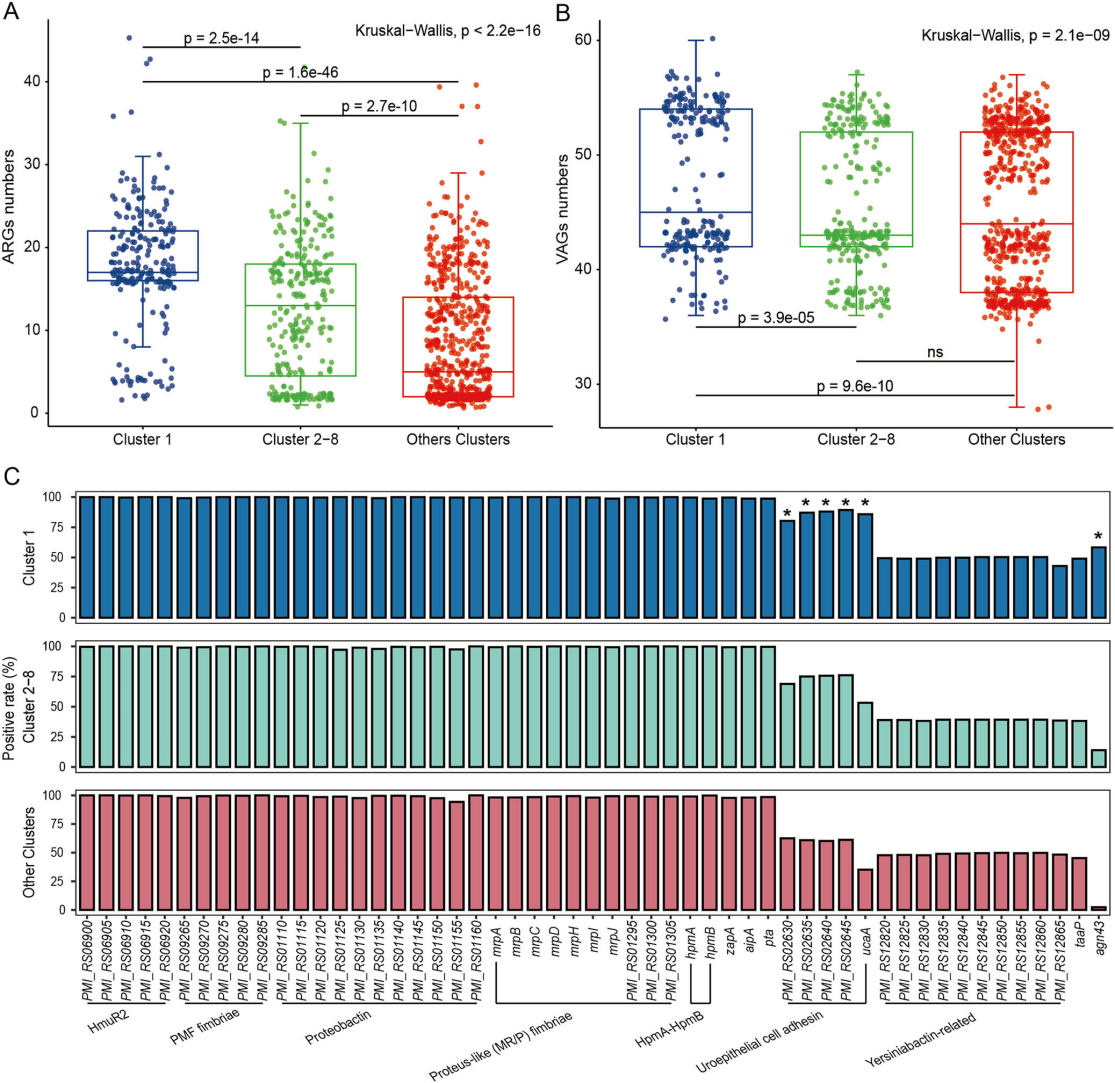
Boxplots comparing total ARG and VAG counts by clusters. **A** ARG carriage by cluster (Pairwise Wilcoxon test with Benjamini–Hochberg adjusted *p* values); **B** VAG carriage by cluster; (Pairwise Wilcoxon test with Benjamini-Hochberg adjusted *p* values); **C** VAG carrying rate by cluster, * Indicates that VAG is significantly enriched in Cluster-1. (Fisher’s exact test)

To facilitate comparison, we stratified the genomes into three groups based on the number of genomes in each cluster: Cluster-1 (major lineage, *n* = 233), Clusters 2–8 (less prevalent lineages, 30–50 genomes in each cluster), and Other Clusters (the remaining lineages, less than 30 genomes). We identified 168 antimicrobial resistance genes (ARGs) in 1142 *P. mirabilis* genomes. Among the three groups, *tet(J)* and *catA* were the most prevalent resistance genes, suggesting that these two genes may be located on the chromosome of *P. mirabilis*. Cluster-1 genomes harbored a significantly higher average number of ARGs compared to other groups (Cluster-1 vs. Clusters 2–8, *p* = 2.5e–14; Cluster-1 vs. Other Clusters, *p* = 1.6e–46) (Fig. 2A). Specifically, Cluster-1 genomes averaged 17.9 ARGs, while Clusters 2–8 and Other Clusters averaged 12.3 and 8.6 ARGs, respectively. A comparative analysis was conducted on the carrying rates of different ARGs in the three groups (Fig. S6). In total, Cluster-1 genomes harbored 102 ARGs, with the carrying rates of 27 of these genes significantly surpassing those observed in the other two groups (*p* < 0.001). Certain ARGs were predominantly found in Cluster-1, including *bla*_KPC-2_ (conferring carbapenem resistance, 41/44), *aadA5* (streptomycin, 108/122), *aac(3)-IId* (gentamicin, 99/123), and *dfrA17* (trimethoprim, 108/122). Additionally, several clinically relevant ARGs, such as the carbapenemase gene *bla*_IMP-27_, extended-spectrum β-lactamase (ESBL) genes (*bla*_CTX-M-65_), and the fosfomycin resistance gene *fosA3*, were also more frequently detected in Cluster-1.

Plasmid replicon analysis showed that plasmids seemed to be relatively rare in *P. mirabilis*, with 66.9% (764/1142) of the strains not having any known plasmid replicon detected (Supplementary Data 1). The most prevalent plasmid replicon was Col3M (254/1142, 22.2%), a small plasmid of 2.6 kbp that encodes a quinolone resistance gene, *qnrD*^10^. This suggests that most of ARGs of *P. mirabilis* may be located on the chromosome.

Furthermore, an investigation into the virulence-associated genes (VAGs) present in the 1142 genomes identified a total of 60 VAGs, categorized into six functional types: nutritional/metabolic factors (*n* = 26), adhesion (*n* = 20), effector delivery systems (*n* = 7), motility (*n* = 3), exotoxins (*n* = 2), and biofilm formation (*n* = 2). 35 VAGs were ubiquitous, detected in over 95% of the genomes, including genes related to iron acquisition (HmuR2, proteobactin), fimbriae assembly (PMF fimbriae, mannose-resistant proteus-like (MR/P) fimbriae), and autotransporter adhesins (*aipA, pta, zapA*). Conversely, four types of VAGs were restricted to a subset of genomes (including urothelial cell adhesin UCA-related genes, yersinib-related genes, trimeric autotransporter adhesin *taaP*, and autotransporter adhesin *agn43*.), and seven VAGs were exceptionally rare, present in only 1 to 3 genomes. Cluster-1 genomes exhibited a modest but statistically significant enrichment in VAGs compared to the other groups (*p* = 3.9e–05 vs. Clusters 2–8; *p* = 9.6e–10 vs. Other Clusters) (Fig. 2B), averaging 47.8 VAGs, while Clusters 2–8 and Other Clusters averaged 45.6 and 45.4 VAGs, respectively. A Fisher’s exact test on VAG carriage rates (Table S2) further revealed significant enrichment of urothelial cell adhesin genes and autotransporter adhesin *agn43* in Cluster-1, with *agn43* displaying the most pronounced enrichment (*p* = 3.8e–68; odds ratio = 22.48, 95% CI: 15.21, 33.67) (Fig. 2C).

To further characterize the distribution of ARGs and VAGs among Clusters 2–8, we analyzed each cluster individually and compared them with Cluster-1. The number of ARGs carried by Cluster-1 was significantly higher than in Clusters 2, 4, 6, and 8, while no significant differences were observed between Cluster-1 and the remaining clusters. For VAGs, Cluster-1 harbored significantly more VAGs than Clusters 2, 3, 5, 6, and 7, but significantly fewer than Cluster 4, and did not differ significantly from Cluster 8. Interestingly, Clusters 4 and 8 carried significantly fewer ARGs than the other clusters, yet these two clusters contained a higher number of VAGs compared with most of the remaining clusters (Fig. S7; *p* values are provided in the Supplementary Data 5 and 6).

**Fig. 3:**
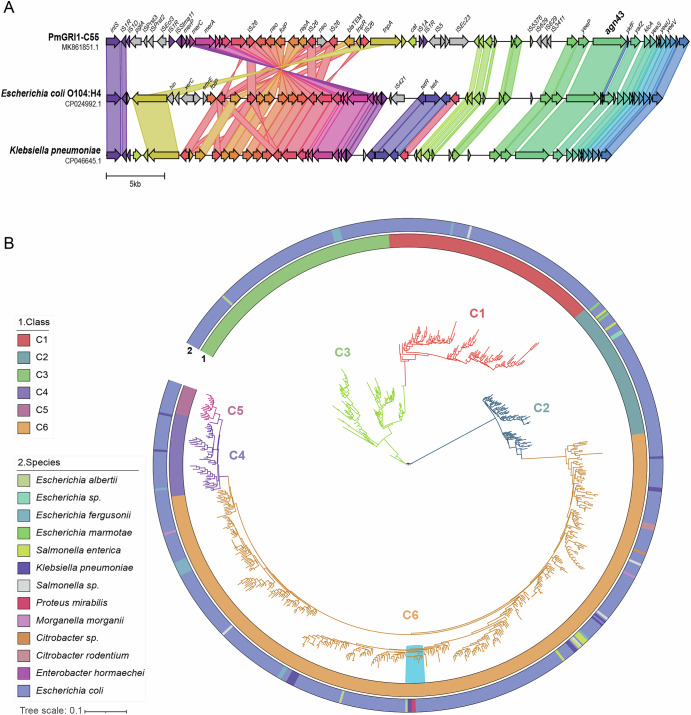
*agn43* is located on PmGRI1. **A** Comparison of the genetic structure of PmGRI1-C55 (*Proteus mirabilis*) and PmGRI1 in *Escherichia coli* and *Klebsiella pneumoniae*. Unannotated genes correspond to hypothetical proteins. **B** Maximum likelihood phylogenetic tree of *agn43*. The light blue block on the tree marks the branch where *agn43* from *Proteus mirabilis* is located.

Our analysis of the genomic resistance island PmGRI1’s genetic architecture revealed that the *agn43* gene resides within this island (Fig. 3A). PmGRI1 also exists in both *Escherichia coli* and *Klebsiella pneumoniae*, exhibiting a high average nucleotide similarity of 98% with *P. mirabilis* (Fig. 3A), implying potential horizontal gene transfer of PmGRI1 and its constituent *agn43*. Analysis of 19 complete PmGRI1 revealed that these genomic islands ranged in size from 19kbp to 155kbp. 36 resistance genes were found on PmGRI1, including 4 genes mediating β-lactam resistance: *bla*_TEM-1_, *bla*_KPC-2_, *bla*_OXA-1_, and *bla*_CTX-M-65_; 1 gene mediating quinolone resistance *aac(6’)-Ib-cr5*; 13 genes mediating aminoglycoside resistance *aac(3)-Ia, aac(3)-IId, aac(3)-Iva, aac(6’)-Ib3, aadA1, aadA16, aadA2, aadA5, aph(3’)-Ia, aph(3”)-Ib, aph(4)-Ia, aph(6)-Id, armA*; 7 genes mediating resistance to folic acid inhibitors were identified: *sul1, sul2, dfrA17, dfrA1, sul3, dfrA7*, and *dfrA27*; 3 genes mediating macrolide resistance: *mph(A), mph(E), and msr(E)*; 1 gene mediating fosfomycin resistance: *fosA3*; 4 genes mediating chloramphenicol resistance: *floR, catA1, catB3*, and *cmlA1*; 2 genes mediating tetracycline resistance: *tet(A)* and *tet(C)*; and one gene mediating rifampicin resistance: *arr-3*. Up to 23 resistance genes were observed on PmGRI1 in a single strain. The most common resistance genes included *sul1, sul2, catA1, bla*_TEM-1_*, aph(6)-Id, aph(3”)-Ib*, and *aph(3’)-Ia*. (Supplementary Data 3) Phylogenetic analysis of *agn43* in *P. mirabilis* specifically pinpointed its classification within the C6 type, exhibiting a single sequence type devoid of variants (Fig. 3B). C6 type is the largest subtype of *agn43* and also the subtype with the most diverse bacterial host sources (including 9 bacterial species). Given that *Escherichia coli* is its main host source, *agn43* in *P. mirabilis* is likely to be acquired from *Escherichia coli*.

### Cluster-1 strains are widely distributed worldwide and can be transmitted across hosts

**Fig. 4:**
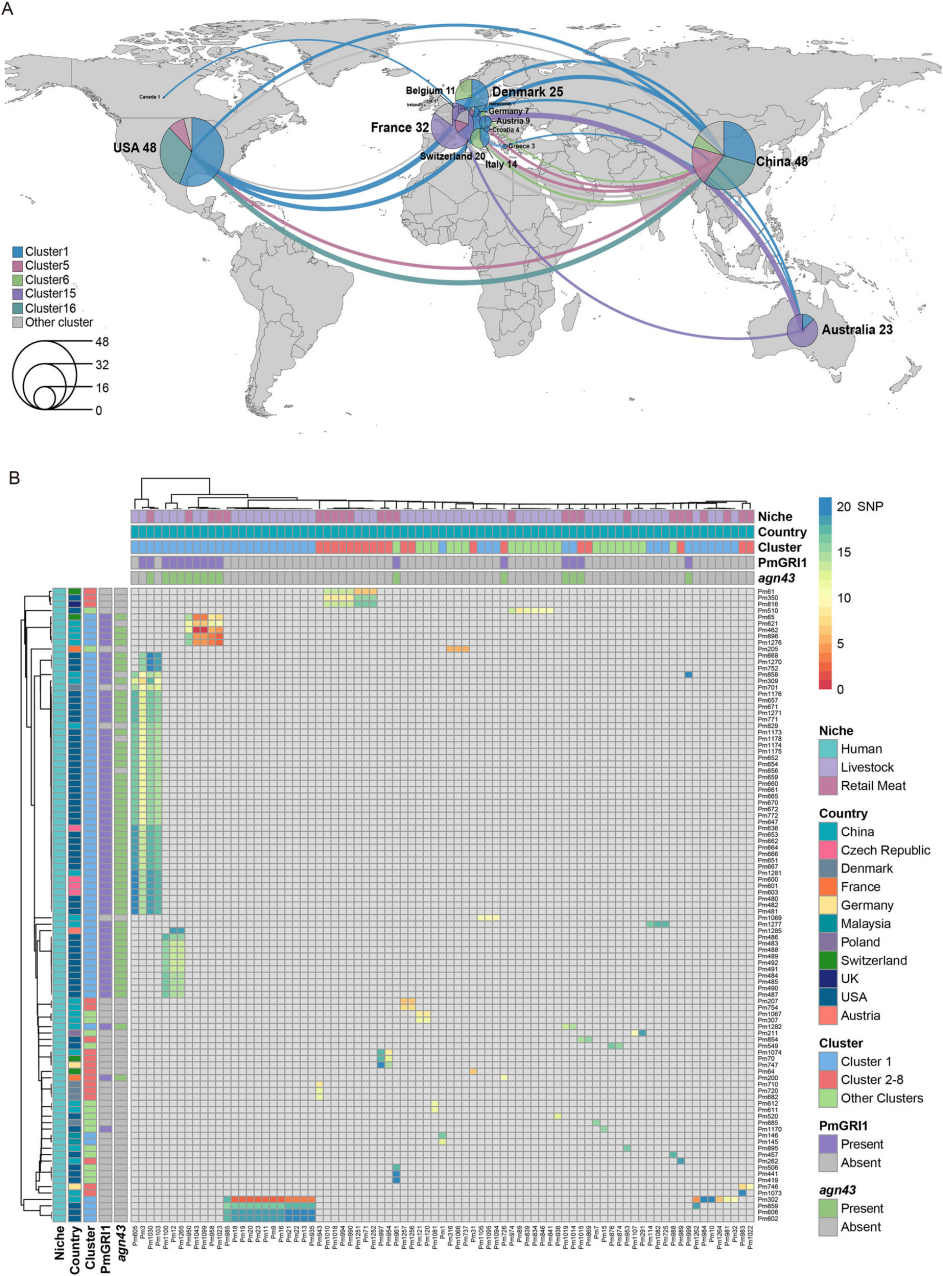
Geographical and host transmission of *Proteus mirabilis.* **A** Transmission of *Proteus mirabilis* between different countries, with 10 SNPs as the threshold. The thickness of the line represents the number of transmission events, and the pie chart shows the proportion of transmission events of different lineages. The larger the pie chart, the more transmission events. **B** Transmission of *Proteus mirabilis* between human and non-human hosts (livestock, retail meat).

To investigate the geographical dissemination of *P. mirabilis*, we quantified pairwise single nucleotide polymorphism (SNP) distances between genomes isolated from different countries. We used thresholds of 5, 10, and 20 SNPs for the analysis. With a 5-SNP threshold, we identified 43 transmission events in total. Cluster-15 (19 events), Cluster-16 (13 events), and Cluster-1 (10 events) were the three major transmission clusters. Using a 20-SNP threshold, we identified 847 transmission events, among which Cluster-1 (664 events), Cluster-2 (41 events), and Cluster-15 (32 events) were the dominant clusters. Employing a 10-SNP threshold, we identified 124 transmission events spanning 16 countries. China and the United States emerged as transmission hotspots, each contributing 48 events, followed by France (32 events), Denmark (25 events), Australia (23 events), and Switzerland (20 events). Among these 124 transmission events, Cluster-1 genomes predominated, accounting for 41.1% (51/124) of all events, indicative of a highly prevalent lineage (Fig. 4A). Within the 51 Cluster-1 transmission events, which involved 11 countries, Denmark–United States represented the primary transmission route (15 events), followed by Australia–United States (9 events) and China–Switzerland (8 events) (Supplementary Data 7).

To further explore potential inter-species transmission, we assessed SNP distances between human isolates and non-human isolates (derived from livestock and retail meat) (Fig. 4B). Our analysis revealed 353 strain pairs within a SNP distance of 20, including 57 pairs within 10 SNPs and 25 pairs within the more stringent 5-SNP threshold. Cluster-1 genomes continued to dominate transmission events between human and non-human strains, accounting for 79.9% (283/354) of strain pairs within 20 SNPs, 73.7% (42/57) within 10 SNPs, and 100% (25/25) within 5 SNPs, highlighting their central role in cross-species dissemination (Supplementary Data 8).

### Phylogenetic dating of Cluster-1

**Fig. 5:**
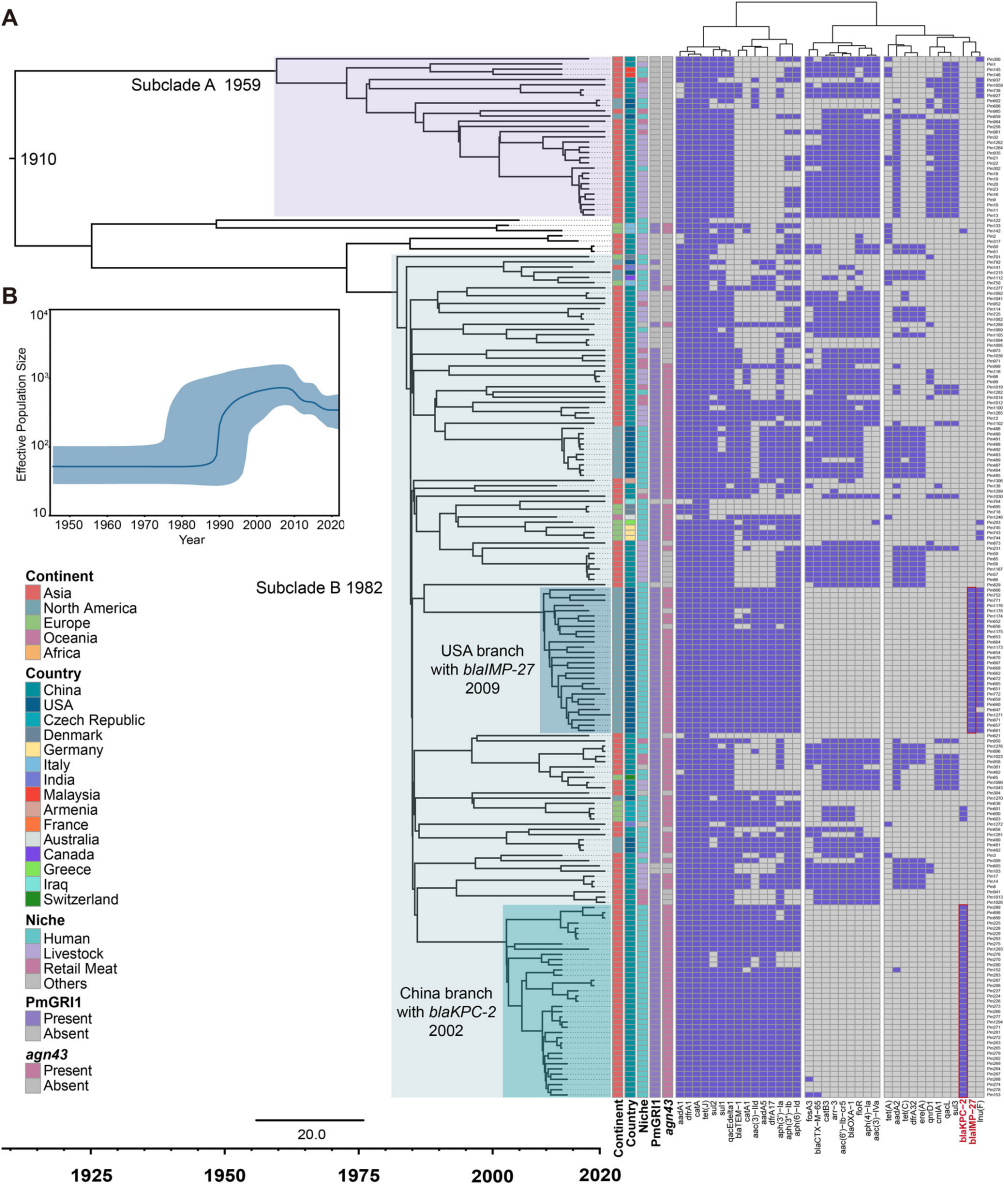
Temporal phylogenetic analysis of Cluster-1. **A** Temporal phylogenetic tree of Cluster-1. **B** Changes in the effective population size of Cluster-1 over time.

To elucidate the temporal origins of the major lineage Cluster-1, we conducted a Bayesian evolutionary analysis of its phylogenetic structure. This analysis encompassed 200 strains of Cluster-1 with precisely documented isolation dates. TempEst analysis showed that there was sufficient temporal signal in the dataset (R^2^ = 0.29, Fig. S8). We tried different model combinations and finally settled on the GTR+strict molecular clock+Coalescent Bayesian Skyline model combination. After executing 100 million Markov Chain Monte Carlo (MCMC) iterations, with sampling every 1000 iterations, we confirmed convergence of the MCMC chains by achieving an effective sample size (ESS) > 200. The median molecular clock rate was estimated to be 3.34e-4 substitutions per site per year (95% CI: 2.27e–4 - 4.43e–4), translating to approximately 0.76 mutations per genome annually. Our analysis estimated the time to the most recent common ancestor (tMRCA) of Cluster-1 to be 1910 (95% CI: 1876-1949). The phylogenetic tree revealed two distinct subclades, Subclade-A and Subclade-B (Fig. 5A). Subclade-A emerged circa 1959 (95% CI: 1939-1982), primarily originating from Asia. In contrast, Subclade-B, which subsequently became dominant, arose in 1982 (95% CI: 1971-1996) and encompassed strains from Asia, North America, and Europe. Notably, within Subclade-B, we identified two evolutionary branches harboring carbapenemase resistance genes: the USA branch, carrying the *bla*_IMP-27_ gene and isolated in the United States (emergence circa 2009, 95% CI: 2006-2013), and the China branch, carrying the *bla*_KPC-2_ gene and isolated in China (emergence circa 2002, 95% CI: 1997-2007). Both branches exclusively comprised human isolates and possessed the PmGRI1. All strains within the China branch harbored the *agn43* gene, whereas two strains in the USA branch lacked this gene. Effective population size analysis revealed a sudden expansion of Cluster-1 in 1989, which coincides with the emergence of Subclade-B (Fig. 5B).

### Effects of the *agn43* on Cluster-1 strains

To investigate the functional impact of the *agn43* gene on *P. mirabilis*, we generated a *agn43* mutant strain, which is belong to Cluster-1.

**Fig. 6:**
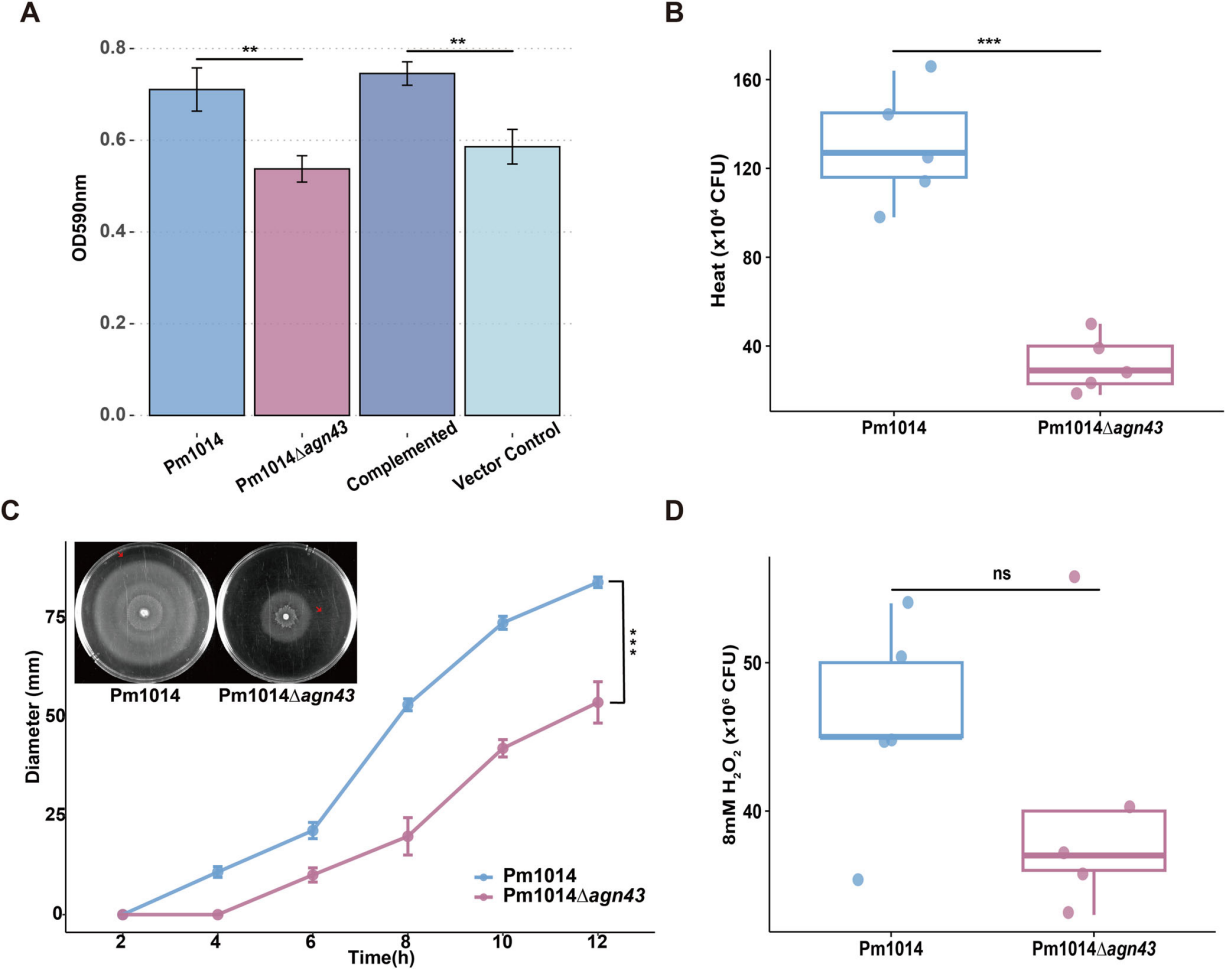
Phenotypic changes of *agn43* mutant strains. **A** Biofilm formation assay. **B** Heat stress tolerance (52 °C, 45 min), colony count. **C** Swarming motility ability assay. The upper left corner shows the 12 h swarming morphology. Since the actual migration edge of the colony cannot be clearly displayed, the actual migration edge of the colony is marked with a red arrow. **D** Treated with 8 mM H_2_O_2_ oxidative stress for 45 min, colony count. *T* test, ns: *p* > 0.05; *: *p* <= 0.05; **: *p* <= 0.01; ***: *p* <= 0.001.

Previous studies have established that *agn43* enhances biofilm formation in *Escherichia coli*. Thus, we initially assessed the biofilm-forming capabilities of *agn43* mutant strain. After 24 h of culture, the biofilm formation of the knockout strain was significantly reduced. After replenishing *agn43*, the biofilm formation returned to the level of the wild strain, as evident in Fig. 6A. We tested the strains’ tolerance to heat (52°C) and oxidative stress (8 mM H_2_O_2_). The knockout strain displayed reduced survival under high temperature stress (Fig. 6B), while no significant differences were observed under oxidative stress (Fig. 6D). Moreover, the mutant strain exhibited a significantly diminished migration rate on LB agar plates (*p* < 0.001), with a noticeable reduction in swarm diameter (53 mm versus 84 mm for the wild-type) after 12 h, as shown in Fig. 6C. However, the heat stress resistance and plate migration ability of the complemented strains were not restored, indicating that the relationship between *agn43* and the above two phenotypes may be indirect.

## Discussion

For a successful epidemic lineage, the development of antibiotic resistance is essential for pathogens to overcome antibiotic treatment and survive in a competitive environment^14,15^. At the same time, several studies have shown that the presence of some virulence genes, the acquisition of virulence plasmids or genomic islands may be genetic determinants for the rapid expansion and spread of pathogens^16–18^. In this study, strains belonging to Cluster-1 harbored an average of 17.9 ARGs, representing a significantly higher genetic burden compared to other lineages. Given the inherent antimicrobial resistance profile of *P. mirabilis* - including natural resistance to polymyxins and tigecycline - the expanded resistome observed in Cluster-1 strains likely confers enhanced survival advantages under antibiotic selection pressure. This selective advantage may partially explain the emergence of Cluster-1 as one of the predominant lineages within the studied population.

On the other hand, virulence gene analysis results showed that urothelial cell adhesin UCA-related genes and *agn43* were significantly enriched in Cluster-1. The UCA-related genes, comprising *PMI_RS02630, PMI_RS02635, PMI_RS02640, PMI_RS02645*, and *ucaA*, are arranged sequentially on the *P. mirabilis* reference genome HI4320^19^. These genes encode proteins involved in the formation of UCA fimbriae, which have been shown to promote binding to urothelial cells and host factors, facilitating urinary tract colonization^1,20–22^. Therefore, strains carrying UCA fimbriae may have an advantage in urinary tract colonization when other fimbriae (such as MR/P fimbriae and PMF fimbriae) are prevalent.

The Ag43 autotransporter encoded by *agn43* enables efficient bacterial aggregation through intercellular self-recognition. In the aggregated state, bacteria are more resistant to a variety of stresses^23^. Another important phenotype associated with Ag43 is biofilm formation. In this study, we found that after knocking out *agn43*, the strain’s biofilm formation were reduced, which is consistent with the functions of Ag43 reported in the literature; the reduced tolerance to heat seems to be explained by the protection provided by Ag43-mediated aggregation, but other mechanisms may also exist. The phenotypic changes in swarming are the most obvious, and its correlation with Ag43 has not been reported. Jones et al. found that swarming motility is essential for migration on urinary catheters, and the multicellular rafts formed by swarming motility enable *P. mirabilis* to migrate from the urethral opening to the bladder through the catheter surface to cause urinary tract infection^4^. Moreover, swarming motility is also considered to be an important means for bacteria to quickly occupy ecological niches^24,25^.

In addition, it is worth noting that the first reported *bla*_KPC-2_-positive *P. mirabilis* in China was isolated from intensive care unit (ICU) of a hospital in Hangzhou in December 2009^26^. Subsequently, in 2010, 19 strains of *bla*_KPC-2_-positive *P. mirabilis* were isolated from ICU ward of a hospital in Hangzhou^27^. Between 2013 and 2019, also in Hangzhou, *bla*_KPC-2_-positive *P. mirabilis* were isolated from several hospitals^28,29^. In 2021, *bla*_KPC-2_-positive *P. mirabilis* was isolated from a hospital in Luzhou, Sichuan, 1400 kilometers away from Hangzhou^9^. All of the above *bla*_KPC-2_-positive *P. mirabilis*, except for strains isolated in 2009 and 2010 (which may not have been uploaded to the public database), are included in the dataset of this study, and all belong to the China branch carrying the *bla*_KPC-2_ gene in Cluster-1. These results indicate that Cluster-1 is a lineage with robust transmission ability and carries clinically important drug-resistant genes. Consequently, close monitoring of the epidemic dynamics of this lineage is warranted to inform effective infection control measures.

This study has the following limitations. First, in the genome-wide association study (GWAS), a total of 86 hypothetical proteins were identified. These hypothetical proteins may have important biological functions, although they are currently uninterpreted, which represents a gap in the research. Second, there are 233 strains in the Cluster-1, while the other lineages only have 30–50 strains or fewer. Although we divided the remaining strains into Clusters 2–8 and Others Clusters to reduce the difference in numbers, combining different lineages for analysis may cause bias in the results, especially when analyzing the number of ARG genes and VAG genes. Third, the phenotype of the *agn43*-complemented strain was not fully restored. One possibility is that the knockout may have affected the transcription level of other genes, and the expression of these genes cannot be directly restored by supplementing *agn43* with a plasmid vector. In situ complementation may be one way to solve this problem.

## Methods

### Bacterial isolates and genome acquisition

A total of 325 *Proteus mirabilis* were preserved in our laboratory from 2008 to 2021, isolated from livestock, humans, and retail meat. The Medical Ethics Committee in Sichuan University approved the protocols (approval number K2022018). The volunteers signed an informed consent form and agreed to the sample collection. Publicly available genomes (*n* = 817) spanning 34 countries and 16 ecological niches (1933–2022) were retrieved from NCBI. Metadata curation included automated retrieval via getNCBImetadata v0.1.0 and manual verification of isolation source/geography. (Supplementary Data 1)

### Genome sequencing, assembly, and quality control

Genomic DNA was extracted using the Tiangen Bacterial Genomic DNA Kit (Tiangen Biotech) and quantified via NanoDrop 2000 (Thermo Fisher Scientific). Qualified DNA samples were sent to Sangon Biotech for Illumina sequencing. The sequencing platform was Illumina Hiseq (paired-end sequencing, 150 bp reads, average sequencing depth 200x). Raw reads were quality-filtered using fastp v0.20.0^30^, followed by de novo assembly using SPAdes v3.15.0^31^. Assemblies were filtered to exclude contigs <200 bp (seqkit v2.5.1^32^) and retained only if meeting quality thresholds (contigs ≤500; N50 ≥ 15 kbp).

### Population genomic and phylogenetic analyses

Core genome alignment was generated using Panaroo v1.3.4^33^(--clean-mode moderate --core_threshold 0.99), with recombination events identified and corrected via ClonalFrameML v1.13^34^ (100 iterations; -emsim 100). A genome-wide association study (GWAS) was conducted by comparing Cluster-1 to all other clusters using scoary v1.6.16^35^ (Benjamini-Hochberg adjusted *p* < 10^–50^). Hypothetical proteins were further annotated using eggNOG mapper v2.1.13^36,37^. A maximum-likelihood phylogeny was reconstructed using IQ-TREE v2.2.5^38^. The phylogenetic tree was visualized using the iTOL online website (https://itol.embl.de/)^39^. Lineage clustering was performed via PopPUNK v2.6.5^40^ (--fit-model lineage -K 4), and visualized using GrapeTree v1.5.0^41^. Multilocus sequence typing (MLST) followed the Proteus scheme^42^ with novel alleles submitted to PubMLST (https://pubmlst.org/).

### Resistance/virulence gene annotation and mobile genetic element screening

The annotation of drug resistance genes and virulence genes was performed using the Resfinder^43^ and VFDB^44^ (https://www.mgc.ac.cn/VFs/Down/VFDB_setB_nt.fas.gz) databases, respectively, with similarity and coverage thresholds of 95% and 85%, respectively. The integrase genes of SGI1, SXT/R391, and PmGRI1 were checked by BLAST to determine the presence of the above mobile genetic elements. Similarly, the Tn*7* integrase *intI2* and *tnsA, tnsB, tnsC, tnsD, tnsE* were checked by BLAST to determine the presence of the Tn*7* transposon. The Tn*7* sequence MK790604.1 was used as the reference. Plasmid replicons were identified using plasmidfinder v2.1.6^45^.

### *agn43* phylogenetic analysis

There are six types of *agn43* genes. In order to determine the type of *agn43* gene carried by *P. mirabilis* and its evolutionary relationship with other types, the *agn43* sequence dataset collected by Ageorges et al. was used^46^. This dataset contains 7336 full-length *agn43* amino acid sequences from 13 bacteria. CD-hit v4.8.1^47^ was used to remove duplicates with a similarity threshold of 0.99. Multiple sequence alignment was performed using mafft v7.520^48^. Finally, IQ-TREE v2.2.5^38^ was used to construct a phylogenetic tree, and the iTOL online website (https://itol.embl.de/)^39^ was used for visualization.

### Bayesian evolutionary dating and phylodynamics

To assess the temporal signal in the ML tree and confirm its reliability, a root-to-tip regression analysis was performed using Tempest 1.5.3^49^ (Fig. S8). Bayesian divergence time inference was performed using BEAST v2.7.3^50^. The site model was set to the Gamma Site Model, the base substitution model was GTR, the molecular clock model was set to the strict molecular clock, the tree prior model was set to the Coalescent Bayesian Skyline, and the Markov Chain Monte Carlo (MCMC) chain length was set to 100 million times, with a sampling interval of 1000 times. The convergence of the MCMC chain was checked using Tracer v1.7.2^51^, and the MCMC chain was considered to have converged when the effective sample size for all parameters was greater than 200. The evolutionary tree was annotated using TreeAnnotator, and 10% of the aging data was discarded. The tree was visualized using FigTree v1.4.4 (http://tree.bio.ed.ac.uk/software/figtree/). The Bayesian Skyline Reconstruction function in Tracer v1.7.2^51^ was used for effective population size analysis.

### *agn43* mutant strain and complemented strain construction

To construct the *agn43* mutant strain, refer to the method of Pellegrino et al. ^1^, the pCVD442 suicide plasmid was used to replace the *agn43* gene on the *P. mirabilis* genome with the gentamicin resistance gene by homologous recombination. Specifically, the upstream and downstream homologous recombination arms of the *agn43* gene were first amplified from *P. mirabilis* Pm1014 (using *agn43*-HA1F/R and *agn43*-HA2F/R primers) (Fig. S8a); the gentamicin resistance gene was amplified from the pJQ200SK plasmid (using *agn43*-GmF/R primers) (Fig. S9a). The upstream and downstream homologous recombination arms of the *agn43* gene were ligated to the Gm resistance gene using fusion PCR to obtain the complete targeting fragment Δ*agn43*::Gm (using *agn43*-HA1F and *agn43*-HA2R primers) (Fig. S9b), which was then cloned into the suicide plasmid pCVD442 to obtain the targeting plasmid pCVD442-Δ*agn43*::Gm. pCVD442-Δ*agn43*::Gm was then transformed into Pm1014 using electroporation. Mutant strains were identified using PCR. The internal primers, *agn43*-inF/R, were designed based on the internal structure of the *agn43* gene; the mutant strains should not produce a band (Fig. S10a). The external primers, *agn43*-outF/R, were designed based on the periphery of the upstream and downstream homologous arms (Fig. S10b). Using Sanger sequencing to confirm that *agn43* had been replaced by a gentamicin resistance gene. The complement strain was constructed using the pUC19 plasmid. The *agn43* gene was cloned into the multiple cloning site of the pUC19 plasmid and then transferred into the mutant strain via electroporation. The complement strain was validated using the *agn43*-pUC19F/R primers (Fig. S10c). Sanger sequencing was used to further verify the sequence correctness of the complement strain. In addition, BLAST was used to verify the specificity of upstream and downstream homologous arms on Pm1014. (The primers used in this experiment are shown in Table S1.)

### Biofilm formation assay

Crystal violet staining was used for semi-quantitative analysis of biofilm formation. Wild-type and mutant strains were streaked on LB plates and cultured at 37 °C until monoclonal formation. Monoclonal clones were picked and added to 3 ml LB solution. They were cultured at 37 °C and 220 rpm until the logarithmic growth phase. After dilution at a ratio of 1:1000, they were added to 96-well plates, with 200 µL per well. To prevent edge effects, the edge wells were filled with 200 µL sterile PBS. The plates were cultured at 37 °C. After culturing for 24 h, the bacterial solution in the 96-well plate was aspirated and discarded, and the plate was gently rinsed 3–5 times with sterile PBS and dried at room temperature; 200 µL of 90% methanol was added to the 96-well plate, fixed at room temperature for 15 min, and the methanol was discarded; 200 µL of 0.1% crystal violet staining solution was added to the 96-well plate, stained at room temperature for 10 min, the staining solution was discarded, and the plate was washed 3 times with sterile PBS; finally, 200 µL of anhydrous ethanol was added to dissolve the crystal violet, and its absorbance at OD590nm was measured using Microplate Absorbance Reader.

### Swarming assay

For the swarming assay, 3 µL of overnight cultured bacteria (wild strain and mutant strain) was pipetted onto the center of the LB plate and incubated at 37 °C. The migration diameter of the strain on the plate was measured every 2 h until 12 h.

### Heat stress tolerance

According to the study by Niu et al. ^52^. Use physiological saline (0.9%NaCl) to adjust the bacterial suspension cultured to the logarithmic growth phase to OD600 = 0.1, dispense 1 ml into 1.5 ml EP tubes and place in a 52 °C water bath for 45 min. Use sterile PBS to dilute 10 times in a row and apply it on LB agar plates for colony counting.

### Oxidative stress assay

Use sterile water to prepare 8 mM hydrogen peroxide solution with 30% H_2_O_2_. Centrifuge 1 ml of the above-mentioned saline bacterial suspension (OD600 = 0.1) at 5000 × *g* for 3 min to obtain bacterial precipitate, discard the supernatant, add 1 ml of 8 mM hydrogen peroxide solution, mix well, and let stand at room temperature for 45 min. Then use sterile PBS to dilute 10 times in a row and apply it on LB agar plate for colony counting.

### Statistics

All statistical analysis were performed in R 4.2.2. The Kruskal-Wallis test was used to determine whether the mean of total ARGs or VAGs per sequence was different based on cluster group membership. Pairwise Wilcoxon test with Benjamini–Hochberg *p* value correction for multiple testing was then used to calculate the significance of the pairwise differences between cluster groups. Fisher’s exact test was used to determine whether PmGRI1 and VAGs were enriched in Cluster-1.

## Supplementary information

Supplementary Information

Supplementary Data 1

Supplementary Data 2

Supplementary Data 3

Supplementary Data 4

Supplementary Data 5

Supplementary Data 6

Supplementary Data 7

Supplementary Data 8

## Data Availability

325 *P. mirabilis* genome sequences in this study have been deposited in NCBI BioProject PRJNA1091058.
