# Supplementary material for: Genomic survey uncovers the emergence of a multidrug-resistant dominant lineage in Proteus mirabilis populations
Source: NPJ Antimicrob Resist. 2026 Mar 3;4:17. doi: 10.1038/s44259-026-00189-5 (PMC12957351; doi:10.1038/s44259-026-00189-5)
Supplement: Supplementary file 1 — Supplementary Information [file 44259_2026_189_MOESM1_ESM.pdf]

Supplementary Data:title and legends

Supplementary Data 1: Dataset, information on 1142 *Proteus mirabilis* genomes.

Supplementary Data 2: genome-wide association study (GWAS), GWAS results.

Supplementary Data 3: PmGRI1, 19 complete PmGRI1 and associated antimicrobial resistance genes.

Supplementary Data 4: Tn7, 349 complete Tn7 elements and associated antimicrobial resistance genes.

Supplementary Data 5: ARG comparison, the p-values of the ARG comparisons for Cluster 1-8.

Supplementary Data 6: VAG comparison, the p-values of the VAG comparisons for Cluster 1-8.

Supplementary Data 7: Cross-Country SNP, cross-country transmission within 20 SNPs.

Supplementary Data 8: Cross-Host SNP, cross-host transmission within 20 SNPs.

Supplementary Figures

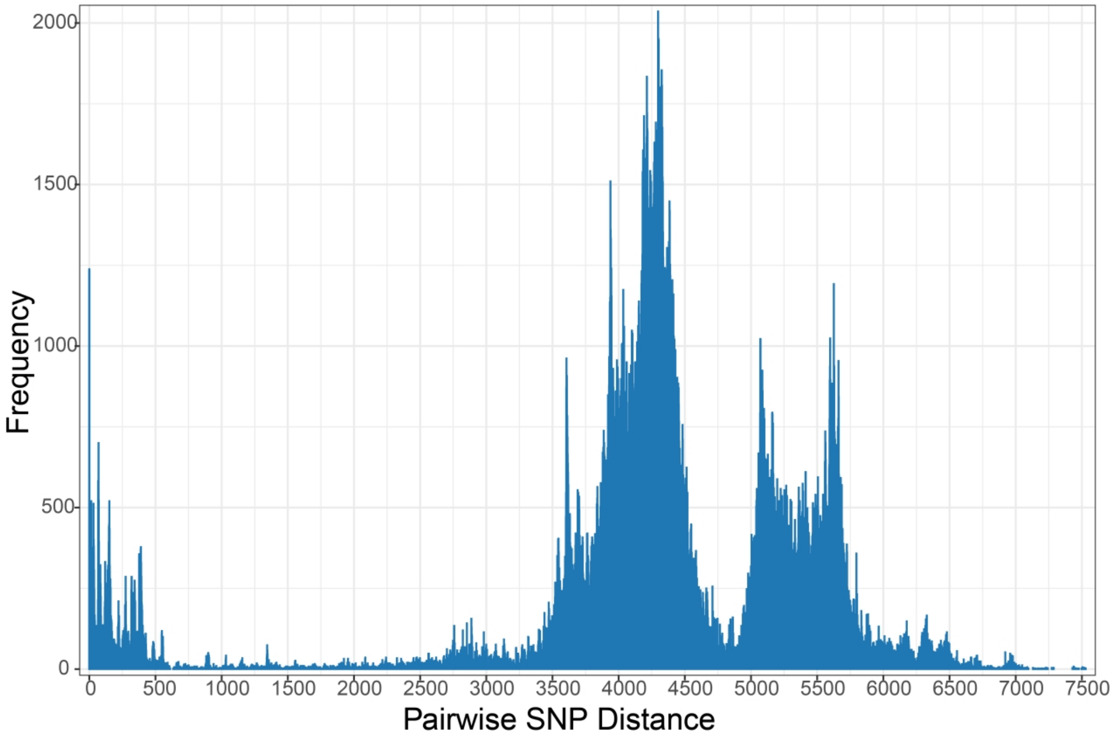

Figure S1. SNP distance distribution of 1142 *Proteus mirabilis* genomes

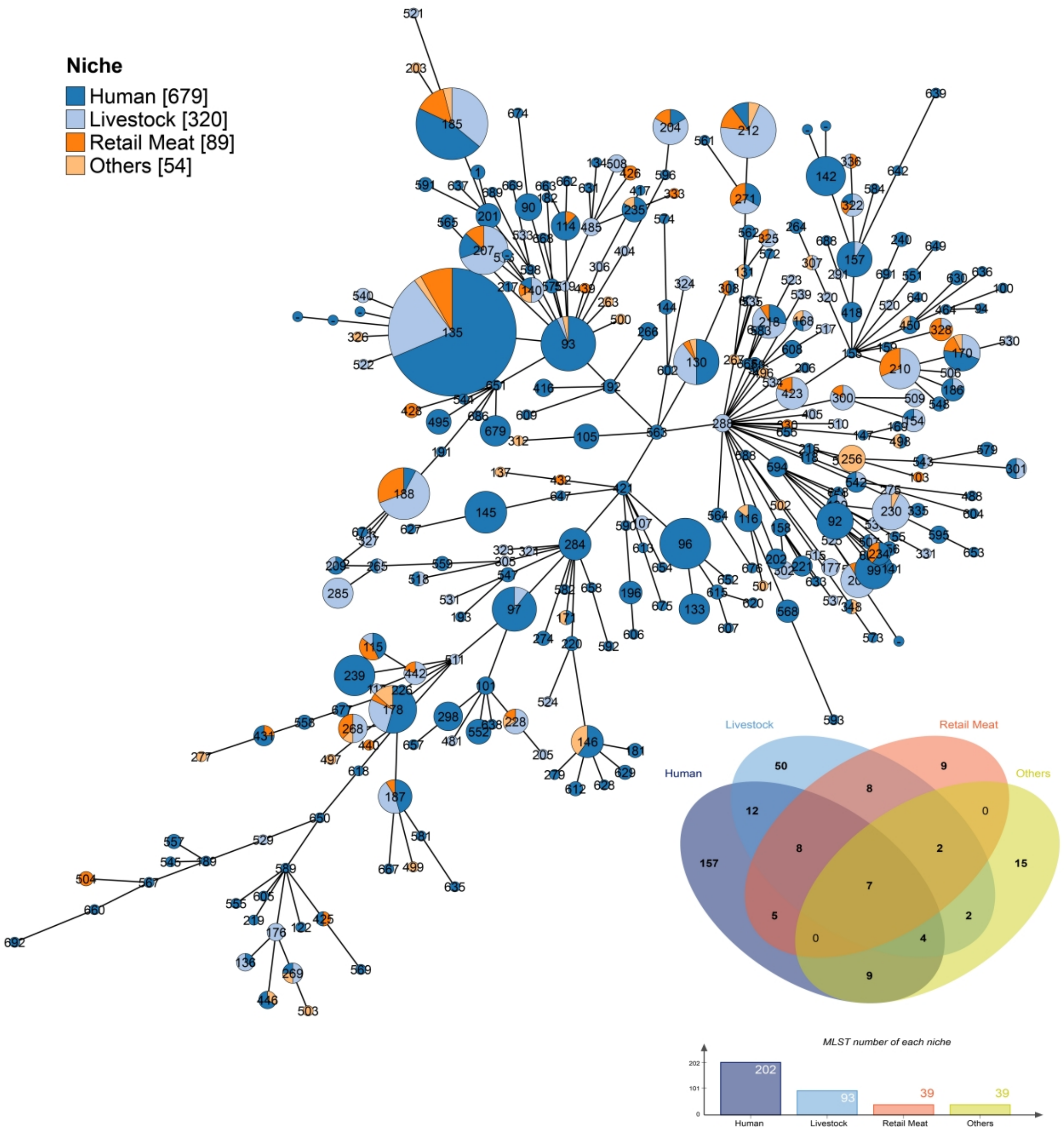

Figure S2. MLST minimum spanning tree of 1142 *Proteus mirabilis* strains (colored by niche). The number in the legend box in the upper left corner is the number of strains in this niche. The Venn diagram in the lower right corner shows the number of ST types that cross between strains from different niches, and the bar chart shows the number of ST types that strains from different niches contain.

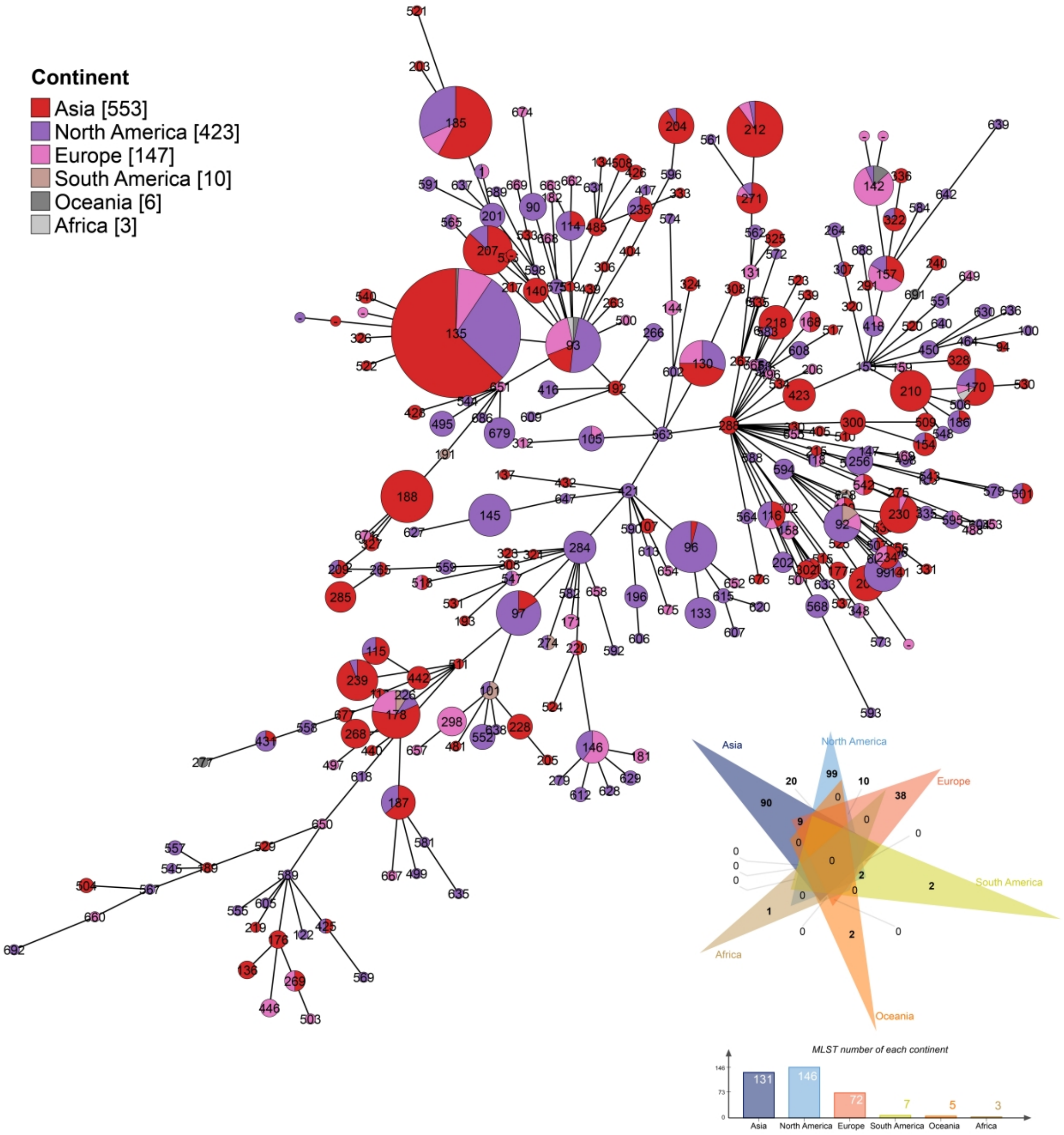

Figure S3. MLST minimum spanning tree of 1142 *Proteus mirabilis* strains (colored by continent). The number in the legend box in the upper left corner is the number of strains in this continent. The Venn diagram in the lower right corner shows the number of ST types that cross between strains from different continents, and the bar chart shows the number of ST types that strains from different continents contain.

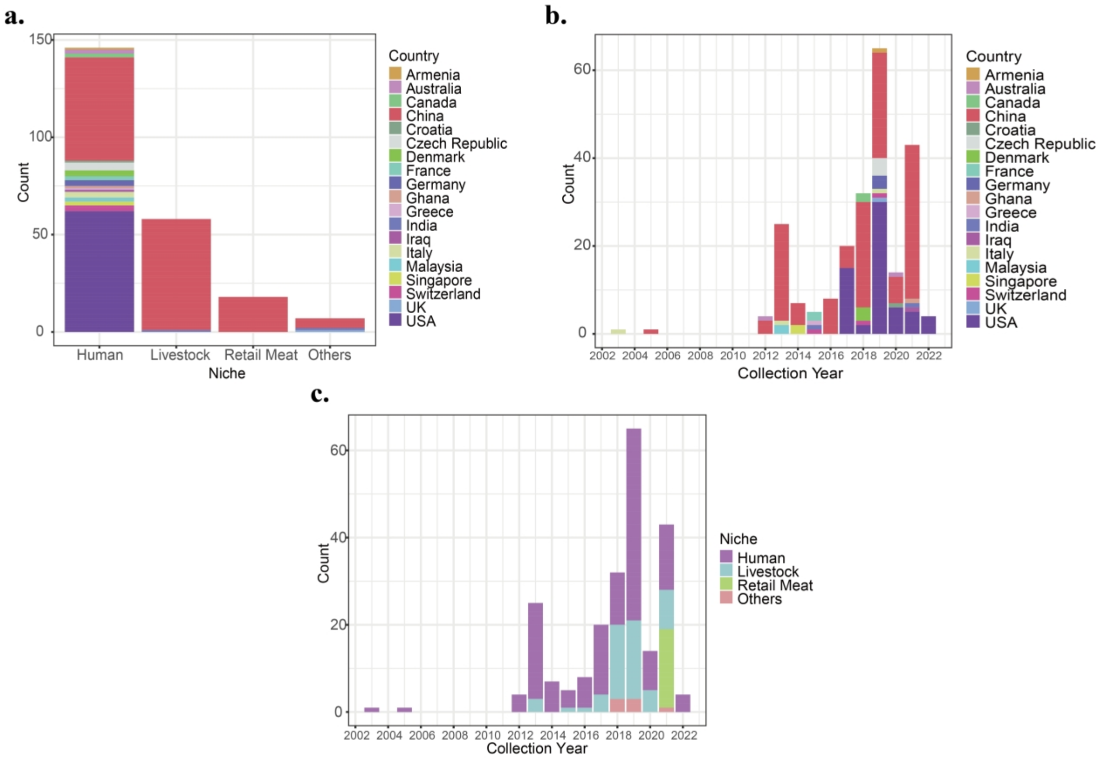

Figure S4. Source distribution of major lineage Cluster-1 strains

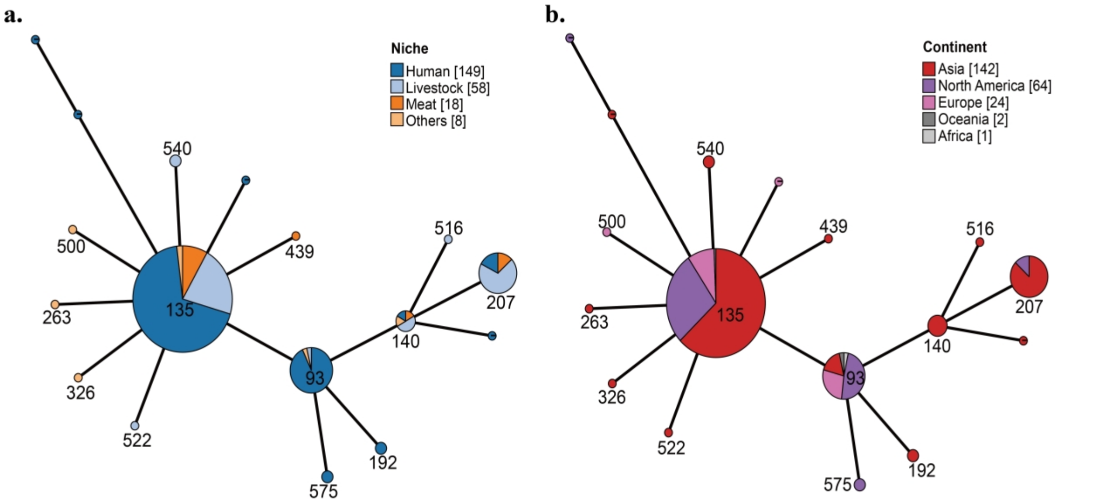

Figure S5. Cluster-1 MLST analysis. Cluster-1 MLST minimum spanning tree, colored by niche. b. Cluster-1 MLST minimum spanning tree, colored by continent.

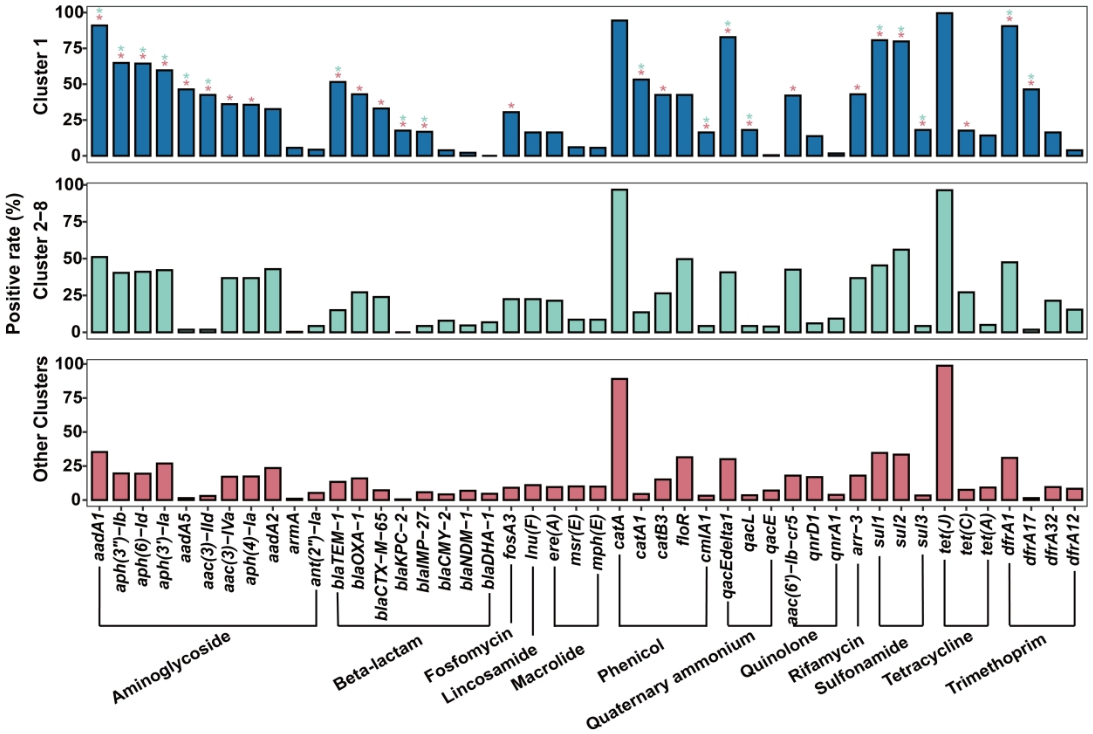

Figure S6. Comparison of ARGs carrying rates in different lineages. Wilcoxon test uses Benjamini–Hochberg corrected p value. The light green * sign indicates that there is a significant difference between Cluster-1 and Cluster 2-8 (adjusted *p*-values < 0.001). The red * sign indicates that there is a significant difference between Cluster-1 and Other Clusters (adjusted *p*-values < 0.001). Only any group of ARGs with a carrying rate of more than 5% is shown on the graph.

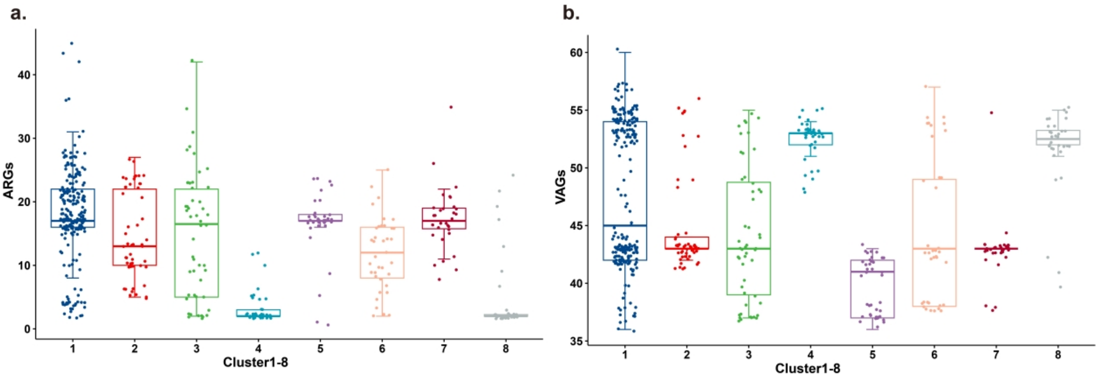

Figure S7. Boxplots comparing total ARG and VAG counts by clusters. **a.** ARG carriage by cluster (Pairwise Wilcoxon test with Benjamini–Hochberg adjusted p-values); **b.** VAG carriage by cluster; (Pairwise Wilcoxon test with Benjamini–Hochberg adjusted p-values). (Supplementary Data)

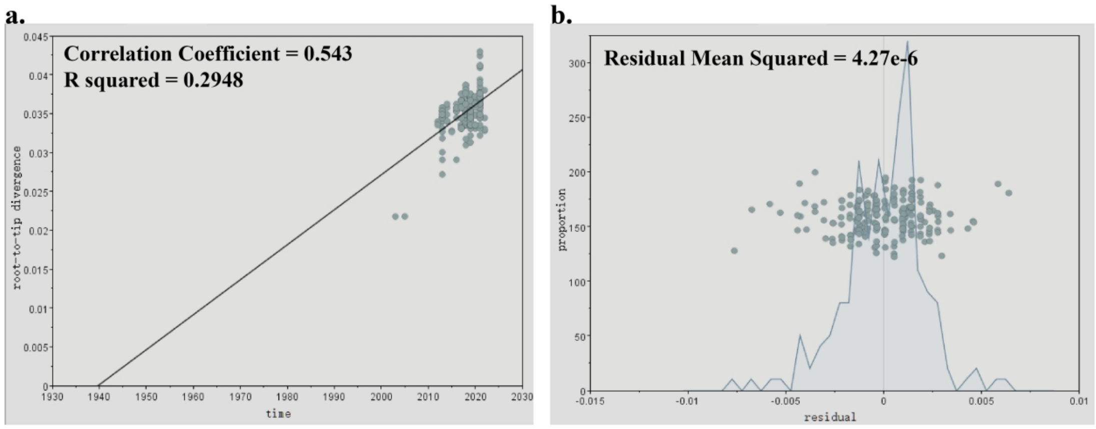

Figure S8. Data set temporal signal detection

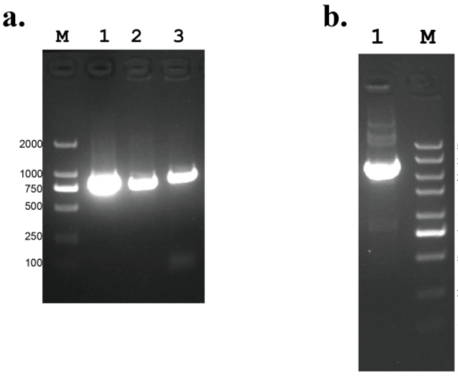

Figure S9. Homologous recombination arm cloning and targeting fragment construction. **a.** 1. Amplification result of gentamicin resistance gene, 874bp; 2. Amplification result of downstream homologous recombination arm, 812bp; 3. Amplification result of upstream homologous recombination arm, 882bp. **b.** 1. Targeting sequence (HA1- GmR-HA2), 2523bp.

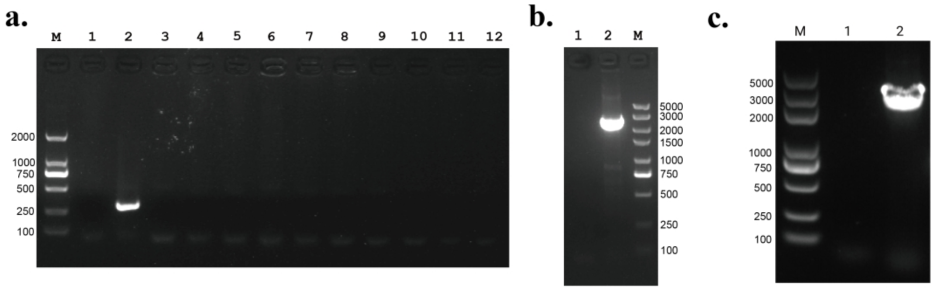

Figure S10. Internal primer identification, external primer identification and complement strain identification. **a.** 1. Amplification results of negative control without template; 2. Amplification results of internal primers of wild strain; 3-12. Amplification results of internal primers of clones No. 1-10 of the mutant strain. **b.** 1. Amplification results of negative control without template; 2. Amplification results of outer primers of clone No. 1. **c.** 1. Amplification results of the empty vector strain. 2. Amplification results of the complement strain.

Supplementary Tables

Table S1. Primer list for gene mutant experiments

primer	sequence（5’-3’）	Length（bp）
agn43-HA1F	GATGAGTATTTCTGGCGACACATCCTG	882
agn43-HA1R	CCTGTAGCTGGTGTTCAGATGTCG	
agn43-HA2F	GGTCAGGCCACGCTGAATGTG	812
agn43-HA2R	CAGAGACTGACCACAGACGCATC	
agn43-GmF	CGACATCTGAACACCAGCTACAGGAGAAATGCCTCGACTTCGC	874
agn43-GmR	CACATTCAGCGTGGCCTGACCTTAGGTGGCGGTACTTGGG	
agn43-inF	CAGATTGTCAAGGAAGGTGGTCTGG	318
agn43-inR	CATGGTGACATCTGTTGCCTTACCAC	
agn43-outF	GTACTGTGGCTGATTAAAGCCGATGAC	2626
agn43-outR	CGTAAGCCCCTTCAATGACTCTGTC	
agn43-pUC19F	ACGGACTGATACTTCTCTGGCG	3011
agn43-pUC19R	CTCGAGTGCGGCCGCAAGCTT	

Table S2. Fisher test on VAGs

VAGs	description	p-value	95 percent confidence interval	odds ratio
agn43	Autotransporter adhesin	3.83E-68	15.20742,33.67060	22.48348
PMI_RS12850	Yersiniabactin-related	0.3392	0.8596302 1.5619852	1.158688
PMI_RS12860	Yersiniabactin-related	0.3392	0.8596302 1.5619852	1.158688
PMI_RS12845	Yersiniabactin-related	0.3043	0.8634317 1.5689227	1.163819
PMI_RS12855	Yersiniabactin-related	0.3035	0.8672503 1.5758943	1.168974
PMI_RS12835	Yersiniabactin-related	0.3039	0.8638065 1.5697369	1.164443
PMI_RS12840	Yersiniabactin-related	0.3395	0.8562192 1.5558697	1.154187
PMI_RS12825	Yersiniabactin-related	0.3383	0.8607389 1.5646020	1.160613
PMI_RS12820	Yersiniabactin-related	0.2392	0.8835322 1.6059053	1.191225
PMI_RS12830	Yersiniabactin-related	0.2692	0.876113 1.592780	1.181413

PMI_RS12865	Yersiniabactin-related	0.5549	0.6704684	1.2246787	0.9070858
PMI_RS02645	Uroepithelial cell adhesin (UCA)	8.44E-14	2.773709	6.989612	4.322353
PMI_RS02640	Uroepithelial cell adhesin (UCA)	5.25E-13	2.585963	6.242689	3.954422
PMI_RS02635	Uroepithelial cell adhesin (UCA)	9.92E-12	2.380734	5.611604	3.602274
ucaA	Uroepithelial cell adhesin (UCA)	2.24E-37	5.916637	13.468897	8.812733
PMI_RS02630	Uroepithelial cell adhesin (UCA)	3.16E-06	1.565126	3.253646	2.239234
taaP	Trimeric autotransporter adhesin	0.1204	0.9407172	1.7111393	1.268865

79
